# Nitrogen use efficiency underlies cross-ecosystem variation in marine primary production

**DOI:** 10.1038/s41598-024-84019-6

**Published:** 2024-12-30

**Authors:** Francis Chan, Karina J. Nielsen, Jane Lubchenco, Bruce A. Menge

**Affiliations:** 1 Department of Integrative Biology, Oregon State University, Corvallis, OR 97331 USA; 2 Cooperative Institute for Marine Ecosystem and Resources Studies, Oregon State University, Newport, OR 97365 USA; 3 Oregon Sea Grant, Oregon State University, Corvallis, OR 97331 USA

**Keywords:** Biogeochemistry, Biooceanography, Climate-change ecology, Macroecology, Ecology, Ecology

## Abstract

The supply of nitrogen (N) and the efficiency with which it is used by phytoplankton serve as two fundamental controls on the productivity of many marine ecosystems. Shifts in nitrogen use efficiency (NUE) can decouple primary production from N-supply but how NUE varies across systems is poorly known. Through a global synthesis of how total N (TN) is apportioned among phytoplankton, particulate, dissolved inorganic, and dissolved organic pools, we demonstrate that NUE underlies broad variations in primary production. Across coastal and open ocean systems, the biomass of autotrophs scales non-linearly with the size of the ecosystem N pool according to a simple equation (chla = 0.004*TN^2.38^) that captures 68% of the variance in chlorophyll-*a* (chla) concentration. Such variation in NUE does not arise from organism-level variation in N-use but reflects ecosystem-level shifts in N-distribution among phytoplankton and dissolved organic-N pools. Because these pools differ in their potential for N-retention, shifts in NUE provide a set of common feedback mechanisms that can act to regulate the long-term stock of N in the surface ocean. Cross-system patterns in NUE provide a set of common relationships for predicting how ocean productivity may respond to future perturbations in N-supply.

## Introduction

The stock of nitrogen (N) in the world’s oceans is apportioned among diverse organic and inorganic pools whose divergent reactivity and residence times govern the global balance of greenhouse gases, productivity of fisheries, and ecological dynamics of microbial and metazoan food webs^1–4^. While the supply of N often sets the ultimate upper constraints to marine production^5^, theoretical predictions also suggest that shifts in the efficiency with which N is acquired and used by primary producers can mediate or decouple the scaling relationship between production and ecosystem N-content (total nitrogen = TN)^6^. In terrestrial ecosystems, conceptual and empirical models have highlighted the importance of resource use efficiency (RUE) in mediating not only productivity but also stoichiometric feedbacks that regulate nutrient cycling, plant community structure, and trophic interactions^7^. Indeed, enhanced RUE through complementarity in nutrient use among species is a central mechanistic conceptual underpinning of biodiversity and ecosystem function research^8^. Nonetheless, in contrast to our understanding of the causal linkages between nutrient supply and primary production in marine ecosystems^9^, the influence of RUE on marine productivity has remained poorly characterized.

In this study, we define nitrogen use efficiency (NUE) as the proportional efficiency with which ecosystem N (TN) is appropriated by autotrophic biomass (N_a_) and available for use in net primary production (NPP). Hence, NUE describes the scaling relationship between N_a_ (as proxied by chlorophyll-*a*) and the total stock of N (TN: sum of all inorganic and organic forms of N) across pelagic systems. If NUE decreases systematically with increasing TN, that would act to dampen cross-system variations in NPP as phytoplankton become less and less efficient at appropriating TN in nutrient-rich systems. Conversely, systematic increases in NUE would accentuate productivity differences between nutrient-poor and nutrient-rich systems. NUE can of course be invariant or vary idiosyncratically across systems, but whether marine production is NUE-neutral or NUE-dependent is not known. Because the supply of N to coastal and open oceans is impacted by direct human alteration of the global N-cycle^10^ and by climate-sensitive changes in circulation and turbulence-driven N-fluxes^11^, understanding how NUE mediates the responses of primary production to shifts in N-supply can provide crucial empirical constraints on our forecasts of future ecosystem change. Despite the conceptual utility of NUE, its application to marine ecosystems has been limited. Two prior studies^12,13^ examined the ratio of chlorophyll-*a* (chla) to TN and total phosphorus with a focus on identifying limiting nutrient(s) across freshwater and marine systems. Using long-term observations from Chesapeake Bay, Denmark Coast, Tampa Bay, and the Dutch Wadden Sea^14^, formally applied the NUE concept to examine system-specific responses to nutrient inputs, and identified non-stationary NUE values that narrow options for eutrophication management. Here, we use a global synthesis of the scaling relationship between N_a_ and TN to test if NUE acts as a common determinant of primary production across diverse coastal and open ocean biomes.

## Methods

### Sample collection

Thirty surface stations on the Oregon inner- to mid-shelves were each sampled 6–10 times from the *R/V Elakha* as part of a study on the spatial distribution of low oxygen zones by the Partnership for Interdisciplinary Studies of Coastal Ocean (PISCO) program. Surface samples were collected by Niskin bottle casts in the upper 5 m of the water column during April and September 2003 spanning the upwelling season. In addition, thirty-eight open coast stations spanning 1,300 km (between 33° N and 46° N) of the United States west coast and 6 stations on the east and west coasts of the South Island of New Zealand (between 44° S and 42° S) were sampled from the shore from May to August (2002 and 2003) and from Oct to March (2002 to 2003), respectively. All sites were directly exposed to the open ocean and were not located within estuaries or embayments and were previously established to examine the ecological dynamics of rocky intertidal communities^15,16^. Sample collection is described in^17^, but briefly, samples were collected by dipping acid-washed 250 ml HDPE bottles into the surf zone. Chla and Particulate nitrogen (PN) samples were filtered in the field onto pre-combusted 25 mm Whatman GF/F glass fiber filters, typically within 30 min, and stored on ice for transport. Samples from New Zealand were held in liquid nitrogen shipping dewar for transport to our laboratory at Oregon State University. The collection and transport of samples in this study were performed in accordance with relevant regulations.

### Sample analyses

Chla was determined fluorometrically^18^ after filters were extracted for 12 h in the dark at – 20 °C in 90% HPLC-grade acetone in a Turner Designs TD700 fluorometer with a detection limit of 0.1 mg and precision of ± 2%. The fluorometer was calibrated against liquid chlorophyll* a* standard from Turner Designs. Dissolved inorganic nitrogen (DIN), the sum of NO_2_^-^ + NO_3_^−^ (N + N), and NH_4_^+^ was determined via flow injection analysis colorimetry^19^ (QuikChem 8500, Lachat Instruments) with detection limits of 0.2 µM, and precision of + /− 5% for both N + N and NH_4_^+^. Particulate organic carbon (POC) and particulate nitrogen (PN) was determined via high-temperature combustion and elemental analysis in a CHN analyzer^20^ (CEC 440HA, Control Equipment Corp), with detection limits of 7 µg and 2 µg for C and N respectively and precision of + /− 0.3% for both analytes. Samples for DIN and POC/PN samples were analyzed at the Analytical Laboratory of the Marine Science Institute, University of California Santa Barbara. Total dissolved organic nitrogen (TDN) was determined via persulfate oxidation and spectrophotometer-based (UV-1201, Shimadzu Corp) colorimetry according to standard methods using EDTA as an oxidation standard^21,22^. Dissolved organic nitrogen (DON) was calculated as the difference between TDN and DIN. Detection limit and precision after accounting for compounding errors in DIN and TDN analyses were 0.4 µM and ± 8%.

### Data compilation and analyses

**Fig. 1:**
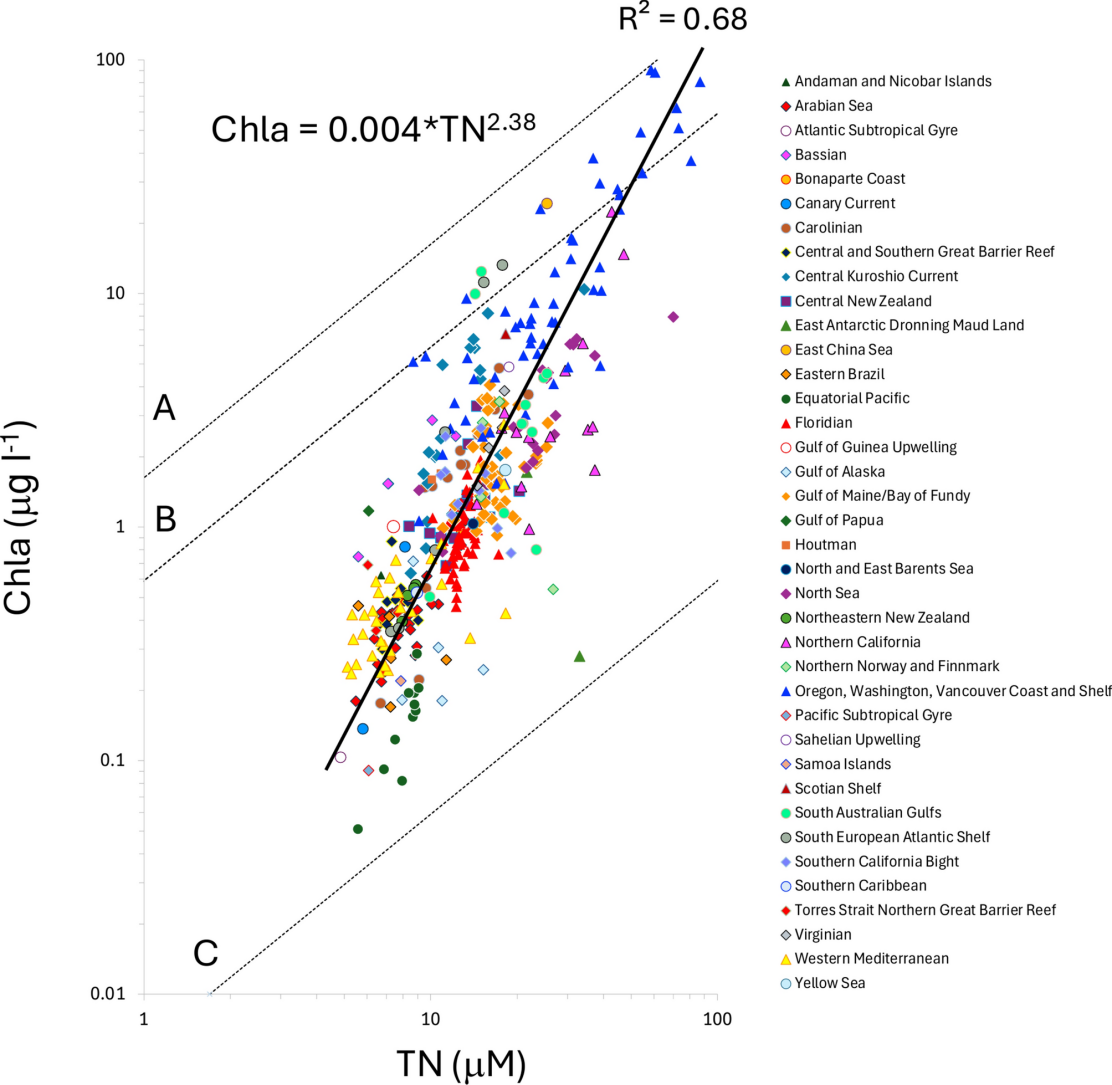
Cross-system pattern of NUE. Reference lines for maximum (100%) and minimum (1%) NUE based on globally averaged C:chla ratio of 48.9 are depicted as A, and C, respectively. Line B reflects maximum NUE if all TN was incorporated as N_a_, but assumes globally-averaged maximum C:chla ratio of 134.8 from ref^39^. Back-transformed orthogonal regression equation yields chla = 0.004* TN^2.54^, n = 391 individual sites. Site means reflect multiple surface layer observations across time.

To increase cross-system coverage, we conducted internet searches for datasets containing observations of chla and total nitrogen (TN), where TN is reported as TN from whole sample digestion or as the sum of total dissolved nitrogen and PN; or DIN, DON, and PN. Not all studies reported or included NH_4_^+^. Because we excluded estuarine samples where anthropogenic inputs of NH_4_^+^ can be substantial, we anticipate that NH_4_^+^ concentrations to be a small portion of the DIN or TN pools consistent with prior studies. For studies that included NH_4_^+^ values in our dataset, the mean concentration was 0.46 µM. This sub-micromolar concentration is consistent with values reported from open ocean and shelf systems^23–25^ but remains a potential source of error in estimates of TN. We did not include any data from estuaries or freshwater systems, restricting our data to only comprise open coast, or open ocean observations to focus our analysis on marine ecosystems where a global synthesis has been lacking. Relaxation of nutrient-limitation, light-limitation and/or top-down control can be temporally dynamic and introduce lags between nutrient supply and primary production. Because we are focused on the mean state of an ecosystem rather than the nature of within system variability, for the relationships between chla, PN, POC, DON, and TN, we included only surface data from stations that were occupied more than once in order to arrive at system means. Where available, surface layer followed the definition of the data source; otherwise the surface layer was defined as the upper 25 m. In total, 391 stations from our field sampling and data compilation efforts spanning coastal and open ocean biomes from 80° N to 70° S (Supplementary Table S1, Supplementary Fig. S1) met the criteria of paired chla and TN observations and temporally repeated sampling. We further derived a maximum NUE reference line based on the Redfield ratio for C:N^26^ and the globally-averaged minimum cellular organic carbon: chla (C:chla) ratio^27^ (Fig. 1). Depth profiles of TN and DIN were poorly represented within the primary dataset of 391 sites. Consequently, we expanded our criteria to include all readily available data of TN and DIN depth profiles regardless of sampling frequency. A complete list of data sources can be found in Supplementary Table S1. NUE was calculated as the yield of chla relative to TN:

1 $${\text{NUE }} = {\text{ chla}}/{\text{TN}}$$

Orthogonal regressions, accounting for error on both independent and dependent axes were used to compute slope and intercepts on log-transformed data where necessary.

## Results and discussion

We synthesized observations of TN and chla that span 170 degrees of latitude representing 37 ecoregions^28^ or 30 large marine ecosystems^29^. Our dataset comprised four orders of magnitude in chla concentration, representing nearly the full dynamic range of phytoplankton biomass recorded for the surface ocean. Global variation in chla shows high correlation (R^2^ = 0.68) with changes in TN (Fig. 1). This scaling relationship between chla and TN is highly non-linear (chla = 0.004*TN^2.38^) and reflects progressive increases in NUE. That is, NUE values from the most TN-rich upwelling systems are as much as 80-fold higher than those from TN-poor oligotrophic gyres. Relative to a maximum NUE value where N_a_ comprises all ecosystem N, observed NUE increases from < 0.5% to > 95% of its maximum potential value along the global TN gradient (Fig. 1). The magnitude of NUE variation indicates that major variation in marine autotrophic biomass does not simply reflect differences in the size of the ecosystem N pool but is subject to broad control by cross-system variation in the efficiency with which ecosystem N is appropriated by phytoplankton. By examining considerably more marine systems, our findings expand from these previous comparative studies^12–14^ to reveal a global scaling relationship between TN and marine production for ocean ecosystems. Across coastal and open ocean ecosystems, chla-poor (rich) systems are always associated with low (high) NUE values (Supplementary Figs. S2, S3). This pattern holds when coastal and open ocean systems are disaggregated (Supplementary Fig. S13) and suggests that NUE underlies general variations in chla in the sea.

What is the basis of this pattern of increasing NUE with increasing TN and chla? Direct mass balance between TN and chla yield (i.e. maximum NUE line that assumes all TN is converted into phytoplankton biomass, Fig. 1) also indicates that for large areas of the open ocean, there is sufficient TN to support much higher levels of chla than is realized. This suggests that shifts in NUE alone have the potential to drive sizeable changes in the productivity of marine systems. Residuals around the regression line indicate that chla can vary by 1 order of magnitude at any specific TN pool size. Such variation can further reflect factors such as top-down control^30^, light limitation^31^, and/or co-limitation by other nutrients^32^. Across a series of well-monitored temperate systems, chla scales as a declining function of TN with exponents ranging from 0.68 to 0.92, possibly as due to phytoplankton self-shading^14^.

It is important to consider if the systematic shift in NUE may in fact be a consequence of nutrient or resource co-limitation that covaries with TN. Iron-limited upwelling regions are marked by high nitrate concentrations, with N-limitation most consistently expressed in subtropical gyre systems^33^. This would suggest high values of NUE in oligotrophic TN-poor (and light-replete) gyre systems and low values in TN-rich upwelling systems where Fe-limitation may constrain production. This prediction is opposite to what was observed in our data (Fig. 1). We do note that chla reported for the Indian Ocean sector of the Southern Ocean, a system where Fe-limitation is prevalent^32^ is markedly low relative to the global regression line (Fig. 1, Supplementary Fig. S2).

Extremely low values of NUE appear to be a pervasive feature of many open ocean systems. Minimum NUE values indicate that in many nutrient-poor systems phytoplankton may be able to use as little as 1% or less of the nitrogen available (Fig. 1). This pattern of declining NUE with the increasing scarcity of N is surprising given the common conceptualization of oligotrophic ocean gyres as systems where available nutrients are readily depleted tightly recycled and held in the biomass of autotrophs. However, our results indicate that such mechanisms do not lead to disproportionate increases in the amount of N held in the biomass of autotrophs in N-poor systems. Alternative N sinks in marine systems include dissolved inorganic nitrogen (DIN), DON, particulate detritus, bacteria, and other heterotrophs. Distribution of N into these alternative pools can mediate phytoplankton success via direct competition for N or indirectly by increasing herbivore biomass and impacts.

**Fig. 2:**
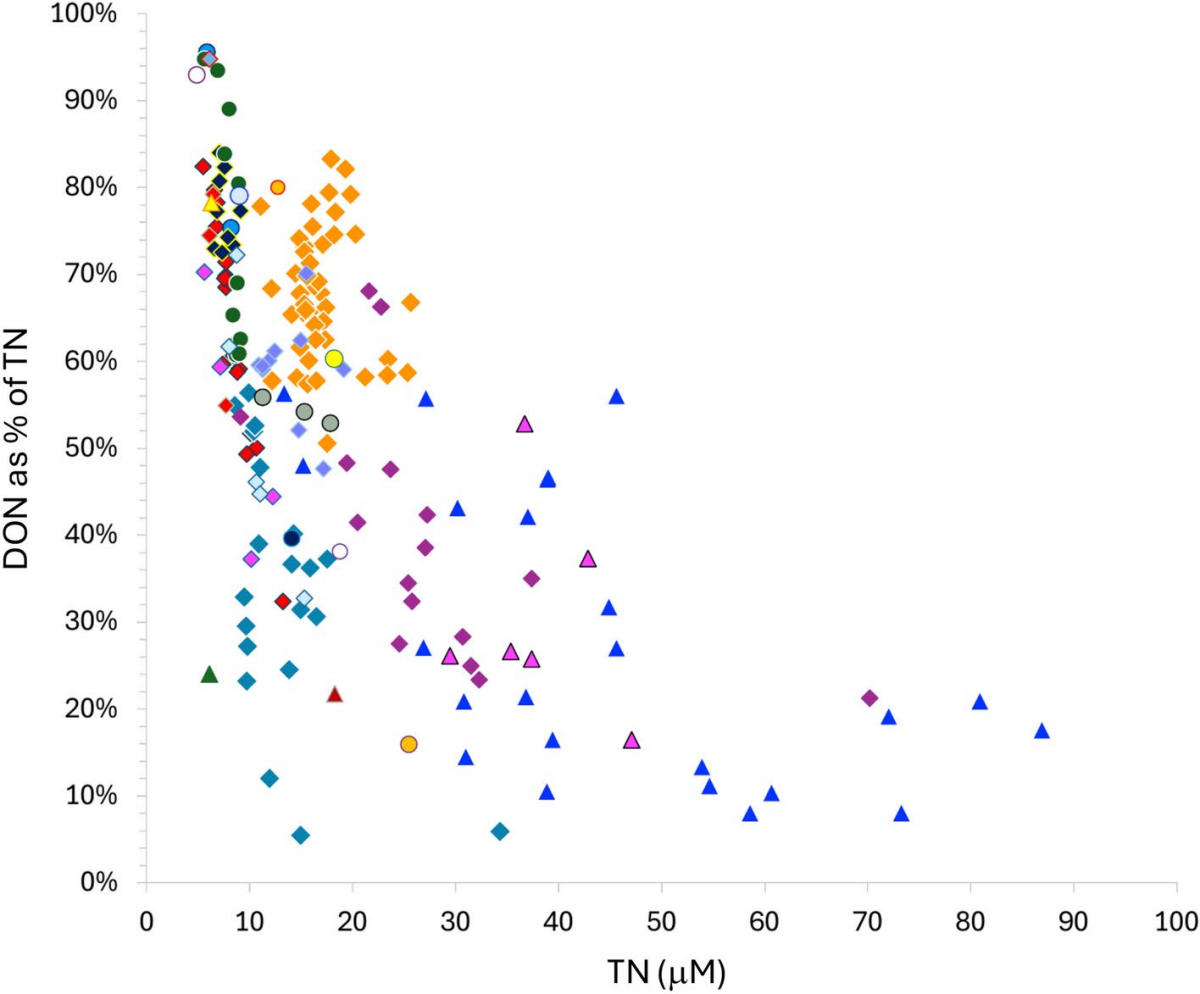
Global pattern in the relative mass dominance of DON along a gradient of increasing TN pool sizes, n = 194 individual sites.

Our assessment indicates that DON progressively dominates the ecosystem N pool as one moves from coastal to open ocean systems and as TN values converge toward global minimum values (Fig. 2, Supplementary Figs. S5, S6). The quantitative dominance of the DON pool has long been recognized in oligotrophic systems^34,35^. Our results indicate that this dominance is strongly dependent on the size of the ecosystem N pool. Although DON uptake can at times, represent a sizeable fraction of N-uptake needs by phytoplankton^35^, the dominance of DON in the least productive systems re-enforces the understanding that the bulk DON pool may be of limited bioavailability^36^.

Extending from the concept of the microbial carbon pump^37^, it has been recently proposed^38^ that the turnover of organic-N through a microbial nitrogen pump results in a progressive accumulation of recalcitrant DON that reduces the amount of labile DON available for recycled production. Observationally, in a transect across 75 degrees of longitude in the South Pacific, rates of primary production varied inversely and strongly with the size of the photic zone DON^39^. This supports the potential for the sequestration of ecosystem N into the DON pool to serve as a fundamental general steady-state constraint on marine production in oligotrophic systems.

The question of bioavailability is important to consider^6^. If DON is viewed as a biologically unavailable pool, then should it be included in the calculation of TN? DON is of course not a single inert pool but comprises pools that vary widely in lability^35,36^. Ideally, TN would include only the portion of the DON pool that is bioavailable. While the characterization of DON lability continues to advance^40^, our quantitative understanding of how DON is apportioned between bioavailable and recalcitrant pools across systems remains limited. In our analysis, we’ve opted to define ecosystem N as the total pool of fixed N that can potentially be available to primary producers. Nonetheless, because DON dominates the ecosystem N pool in oligotrophic systems (Fig. 2), it is important to assess the effects of excluding DON. We find that the exclusion of DON from the TN pool (Supplementary Fig. S7) does not reverse the pattern of increasing NUE with TN (Fig. 1, Supplementary Fig. S2). Chla increases as a non-linear function of PN + DIN (Chla = 0.084* PN + DIN^1.65^, R^2^ = 0.71), such that the ratio of chla:PN + DIN in the most oligotrophic system is 4 orders of magnitude lower than that for the most nutrient-rich systems (Supplementary Fig. S7). While the pool of recalcitrant DON likely serves as a general upper constraint to NUE, the variations in DON concentration within and across ecosystems suggest that the degree to which DON serves as a sink or source for autotrophic N is a property of individual systems. The inclusion of DON in the ecosystem N pool provides a means to measure that property.

Our assessment of NUE uses chla concentration as an index of phytoplankton biomass (N_a_) and net primary production (NPP). What is the justification for this decision? Because chla:N_a_ varies as physiological and phylogenetic responses to changing environmental conditions^41^, variations in NUE may simply reflect physiological shifts at the organism-scale rather than ecosystem-level changes in N appropriation by autotrophs. Our maximum NUE line (Fig. 1) is calculated from the commonly measured cellular organic carbon: chla (C:chla) ratio and the Redfield C:N ratio, where C:chla reflects the minimum globally-averaged value (48.9) reported in^27^. A secondary reference line that is based a maximum globally-averaged C:chla value of 134.8^37^ serves to bound the effects of chla:N_a_ variation. The C:chla can vary considerably depending on system and physiological state of phytoplankton^42,43^. Assuming a lower (higher) C:chla ratio as might be encountered in nutrient-replete and/or light-limited (nutrient deficient and/or light-saturated) cells^41^ would act to move the maximum NUE line higher (lower). Thus, the two reference lines A, B, (Fig. 1, Supplementary Fig. S8) provide a bound for the effects of a C:chla ratio range of 85.9. The vast majority of chla values fall outside this range indicating limits of organism-scale shifts in chla:N_a_ stoichiometry to account for cross-system variation in NUE.

**Fig. 3:**
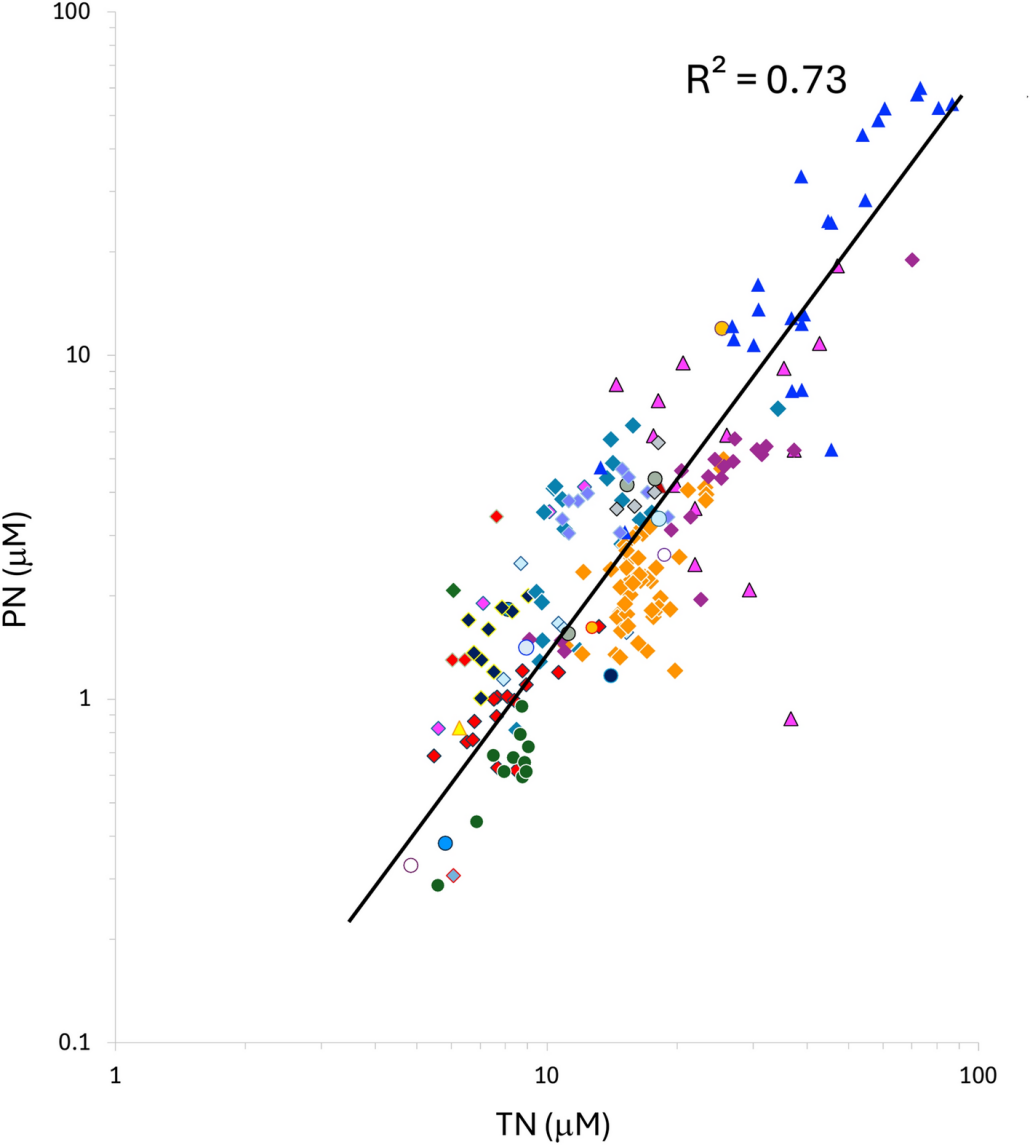
Particulate nitrogen (PN) and total nitrogen (TN) across systems. Back-transformed orthogonal regression equation PN = 0.027 TN^1.69^, n = 205 individual sites.

Direct measures of N_a_ would of course sidestep the need for stoichiometric assumptions but N_a_ is notoriously difficult to ascertain, requiring separation of phytoplankton cells from other particulate materials^44^, and are currently unavailable for cross-ocean biome comparisons. Besides chla, bulk particulate nitrogen (PN) may provide an alternative index of N_a._ However, PN comprises detrital and heterotrophic N in addition to autotrophic N. Nonetheless, PN does serve as an upper constraint on potential N_a_. For example, across systems, PN and chla are strongly coupled (R^2^ = 0.82) with the chla: PN ratio increasing by approximately 1 order of magnitude across the range of observed values (Supplementary Fig. S9). This shift is consistent with previously documented increases in the ratio of autotroph: heterotroph biomass along marine productivity gradients^45,46^. Because PN and chla are tightly coupled (Supplementary Fig. S9), like chla, the relative size of the PN pool also increases non-linearly along the TN gradient (Fig. 3).

A second assumption in our analysis is the utility of chla in indexing NPP. This reflects the strong empirical relationship between the standing stock of chla and NPP across open ocean and coastal biomes^27,47^. Nonetheless inferring flux from stock is subject to uncertainties in how phytoplankton turnover rates vary across systems, and NPP is most robustly modeled when environmental factors such as photosynthetically active radiation, mixed layer depth, and temperature accompany chla estimates^48^. The lowest values of NUE were found in subtropical oligotrophic ocean gyres where the dominance of phytoplankton biomass by picoplankton^49^ would suggest the potential for phytoplankton turnover rates to compensate for reduced chla. The 4 orders of magnitude of differences in chla described here would require phytoplankton in low chla systems to turnover at rates that are 4 orders of magnitude faster to equal the productivity of high chla systems. Such rates were not documented in in situ phytoplankton growth rate measurements across systems^50^. Broad scale cross-biome surveys report turnover rates that vary by only 2 to 4 folds, often with the highest rates encountered in productive, chla-rich systems^51–53^. These observations suggest that potential turnover rate differences alone cannot explain the globally divergent patterns in NUE.

Does NUE arise from uptake or production efficiency? Mechanistically, nitrogen use efficiency must ultimately scale from the (1) efficiency with which available N is acquired by autotrophs (uptake efficiency), and (2) rate of NPP per unit of acquired N (production efficiency)^54^. Production efficiency can be particularly important in terrestrial systems where acquired N is used repeatedly to support the fixation of carbon into N-poor woody biomass^55^. In aquatic short-lived autotrophs under balanced growth, tissue C: N approximates the ratio of C to N uptake^56^, and can be used as a measure of production efficiency. Deviations in NUE due to production efficiency would thus manifest as departures from the Redfield ratio in particulate organic-C (POC) and PN. This ratio can exhibit marked within-region variation^57^. But globally, POC: PN was highly conserved in our dataset, showing strong adherence to the Redfield ratio across the 4 orders of magnitude variation in particulate organic matter concentration (Supplementary Figs. S10, S11) and consistent with previous cross-system compilations^58^. These patterns suggest that like chla: N_a_, variation in NPP:N_a_ as proxied by POC:PN is relatively conserved at the cross-biome scale and is unlikely to serve as a determinant of NUE variation in our analysis.

Deviations in phytoplankton production efficiency can also theoretically arise from the extracellular release of dissolved organic carbon (DOC) and/or high C:N compounds. For example, exudation of extracellular DOC comprises on average ~ 20% of NPP, though in oligotrophic systems, extracellular DOC release can comprise up to 50% of NPP^59^. With respect to C:N, the mean ocean surface C:N ratio of bulk dissolved organic matter does not deviate strongly from the Redfield ratio^5^, averaging 14.9:1 globally^60^. These observations suggest extracellular DOC releases or non-Redfield ratio of dissolved organic matter releases alone, may be insufficient to give rise to sufficiently variable production efficiency values given the 4 orders of magnitude variation we observed in chla (Fig. 1).

**Fig. 4:**
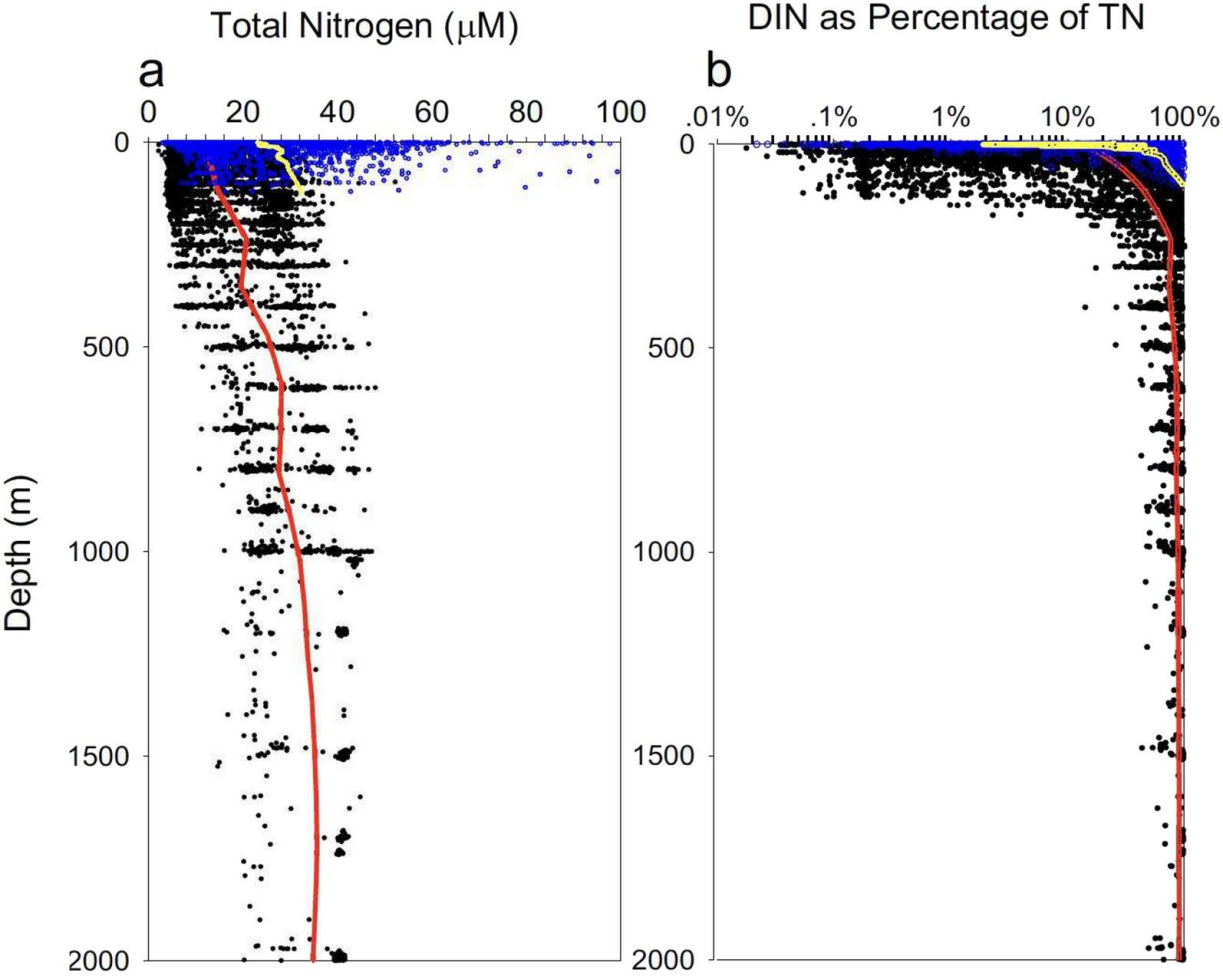
Depth profiles of TN (**a**) and DIN:TN (**b**) to 2000 m across systems. Coastal blue circle and open ocean black circle profiles include additional sites that were sampled only once (Supplementary Fig. S1). A total of 3184 individual profiles are represented. LOWESS regression lines are depicted separately for coastal yeollow line and open ocean red line sites.

The size of the ecosystem N pool is surprisingly conserved across a diverse array of marine biomes (Fig. 4a). Coastal sites exhibit greater mean and range than open ocean sites, but the central 90% of all surface TN observations fell between 6.4 and 37.2 µM. Because DIN comprises a major portion of the TN in deep waters (Fig. 4b), mixing and transport can drive increased N-availability in general. This increase in the supply of readily useable N by phytoplankton thus is likely to contribute to the trend towards increased NUE in more TN-rich systems. The absence of TN endmembers > 40 µM in waters of intermediate depth (150–500 m) suggests that high coastal TN values can further reflect processes such as terrestrial inputs^10^ and/or nutrient accumulation^61^ on continental shelves.

The relatively narrow distribution of TN values (Fig. 1) suggests that the size of the marine ecosystem N pool may be subject to strong feedbacks that act to constrain the depletion as well as accumulation of N in surface waters. Our observations reveal a systematic trade-off between the dominance of phytoplankton and DON pools along the TN gradient (compare Figs. 1, 2 and 3). This trade-off offers one general mechanism for regulating ecosystem N pool size in the ocean. Since the sinking of particulate organic matter represents the principal loss of N from surface mixed layers, the increased sequestration of N into phytoplankton biomass, and shifts toward larger, more sedimentation-prone phytoplankton taxa in more productive systems^62^ would serve to constrain the long-term accumulation of N in high TN waters. The reductions in NUE and dominance of DON—a pool not subject to direct sedimentary losses in oligotrophic systems—can act in an opposing fashion to regulate the loss of N from surface waters and set the lower limit to the size of the TN pool. These feedbacks suggest that NUE may act as a key feature coupling the internal biogeochemical structure of marine ecosystems with the regulation of the size of N pools in the surface ocean.

In terrestrial systems, NUE is often conceptualized as declining along gradients of increasing ecosystem fertility^6,63^. This pattern arises because mechanisms that act to conserve the loss of limiting resources such as selective resorption of N from senescing leaves, and/or maximize production efficiency, relax with increasing nutrient availability^7,54^. A reasonable expectation is that analogous mechanisms such as the ability to draw down DIN to exceedingly low concentrations or tight retention and recycling of N in oligotrophic marine systems would also act to maximize NUE. In contrast, marine NUE is lowest in nutrient-poor systems such as sub-tropical gyres and highest in nutrient-rich systems such as upwelling shelves (Fig. 1). This suggests that high uptake affinities for DIN or closed N-cycle associated with oligotrophic systems are insufficient for maximizing NUE at the ecosystem scale. As noted above, NUE is the product of production efficiency in the rate of NPP per unit of N_a_ and N uptake efficiency in the acquisition of N from the environment. Consequently, cross-system differences in NUE can be driven by shifts in the relative importance of production and uptake efficiencies. In terrestrial systems, where long-lived plants exhibit substantial internal recycling of N, production efficiency can vary widely and play a key role in modulating NUE along gradients of N-fertility. For pelagic systems considered here, nutrient uptake efficiency is likely to play the dominant role in determining cross-system variation in NUE.

The mechanism(s) that drive the progressive increase in NUE and uptake efficiency with TN cannot be resolved by our correlative study. Shifts in the strength of top-down control of phytoplankton by consumers along the microbial vs. metazoan food web continuum^64^, or transient increases in DIN availability in upwelling and seasonally destratified systems^65^ are alternative processes that can structure and reinforce the ability of autotrophs to effectively appropriate ecosystem N. These represent logical next steps in bringing a mechanistic understanding to controls on NUE across systems.

Future efforts that expand TN and chla observations to systems where limitation or co-limitation by other nutrients will be important to test the stability of the global scaling relationship. In particular, large regions of the ocean are limited by iron but replete in nitrogen. Prominent high-nutrient, low-chlorophyll (HNLC) systems include the Southern Ocean, Northeast Pacific, and Equatorial Pacific upwelling. While these systems are represented in our dataset, the number of observations is limited relative to other coastal and other oceanic sites. As systems that are essentially defined by having low NUE values, a comprehensive representation of HNLC systems would be invaluable. The stability of NUE in time also deserves attention. Carstensen et al.^14^ found strong evidence of non-stationarity in NUE within individual coastal estuarine systems potentially in response to alterations in nutrient inputs, food web dynamics or climate change.

Our results indicate that in marine ecosystems, realized variation in NUE can impose fundamental constraints on productivity. While the dependence of pelagic production on ecosystem nutrient pool size in freshwater ecosystems is a central tenet of predictive limnology^66,67^, our results highlight a similarly important and globally pervasive coupling between nutrient pool size and chla across marine ecosystems. Despite the heterogeneous nature of marine biomes, our analyses reveal common responses of marine biogeochemical structure to changes in the size of the ecosystem N pool. The linkage between TN and NUE further indicates incremental changes in TN can result in strongly non-linear responses in marine primary production. Accelerating inputs of anthropogenic N^10,68,69^ and climatically-driven shifts in transport and mixing^11,70^ are central perturbations to the N-cycles of marine ecosystems. For many marine systems facing reductions in nitrogen supply from warming waters, the global functional relationship between TN and NUE suggests the likelihood for empirically-constrained nonlinear declines in primary production.

## Supplementary Information

Supplementary Information 1.

Supplementary Information 2.

## Data Availability

The datasets generated during and/or analyzed during the current study are available at: https://github.com/FChanOSU/NUE.
