# Supplementary material for: Nitrogen use efficiency underlies cross-ecosystem variation in marine primary production
Source: Sci Rep. 2024 Dec 30;14:32146. doi: 10.1038/s41598-024-84019-6 (PMC11685585; doi:10.1038/s41598-024-84019-6)
Supplement: Supplementary file 1 — Supplementary Information 1. [file 41598_2024_84019_MOESM1_ESM.pdf]

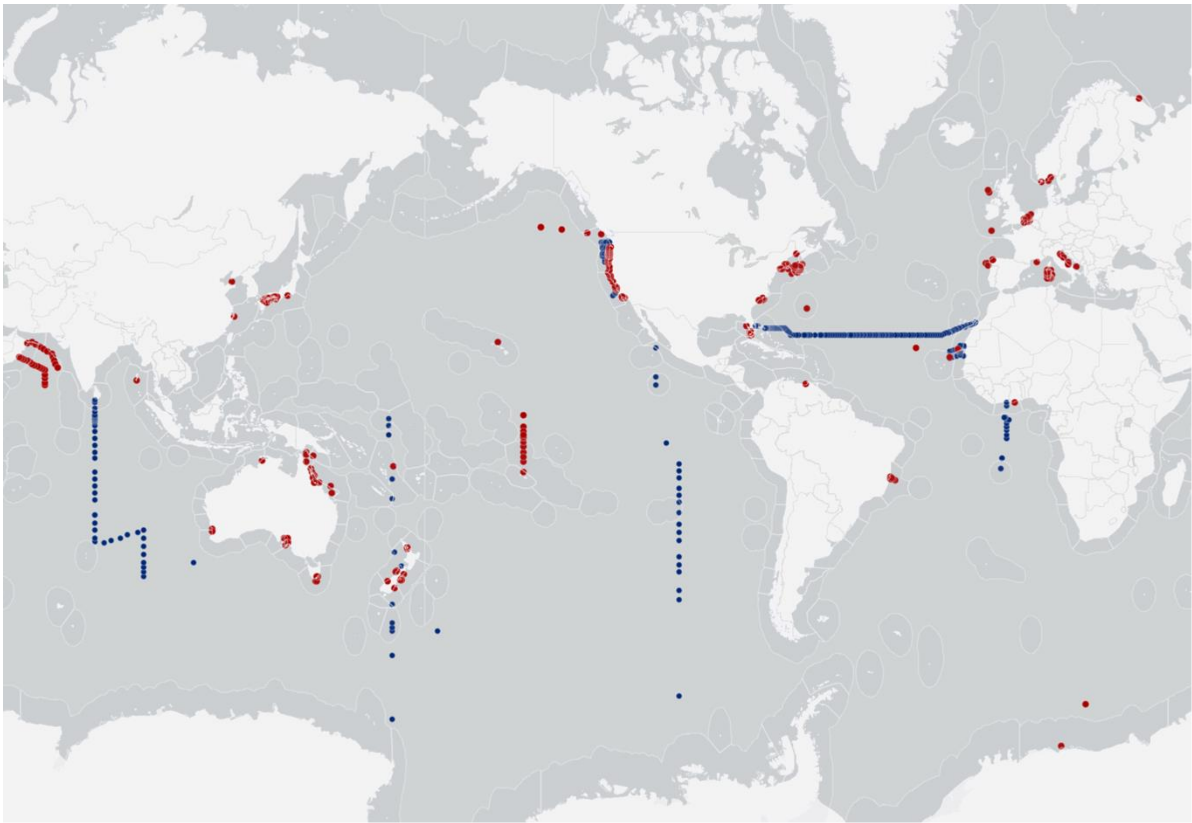

Figure S1. Locations of data source used in this study. Core stations for NUE analysis (n = 391) and depth profiles are depicted in red. Additional single visit stations (n = 502) included in TN and DIN:TN depth profiles are depicted in blue. Marine ecoregions as defined by Spalding et al. 2007 are outlined. We aggregated sites by ecoregions (Fig S3, S5) to reduce leverage by highly sampled systems and because large marine ecosystems are restricted to coastal ocean environments.

Spalding, M.D., Fox, H.E., Allen, G.R., Davidson, N., Ferdaña, Z.A., Finlayson, M.A.X., Halpern, B.S., Jorge, M.A., Lombana, A.L., Lourie, S.A. and Martin, K.D., 2007. Marine ecoregions of the world: a bioregionalization of coastal and shelf areas. *BioScience*, *57*(7), pp.573-583.

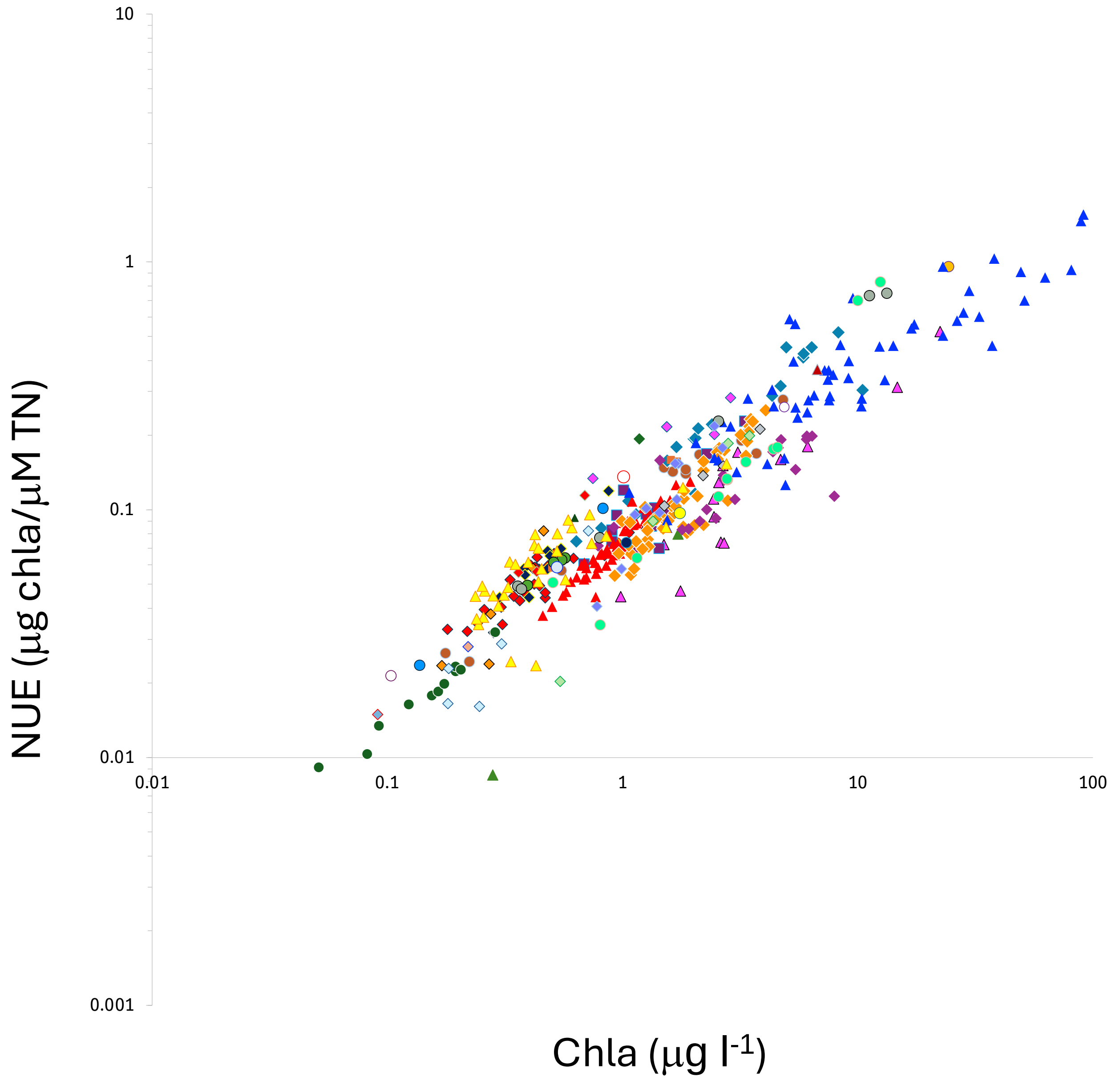

Figure S2. NUE variation along gradients of Chla illustrating the strong association between these two ecosystem properties such that chla-poor but high NUE or chla-rich but low NUE are not observed states in the ocean. Site symbols follow figure 1.

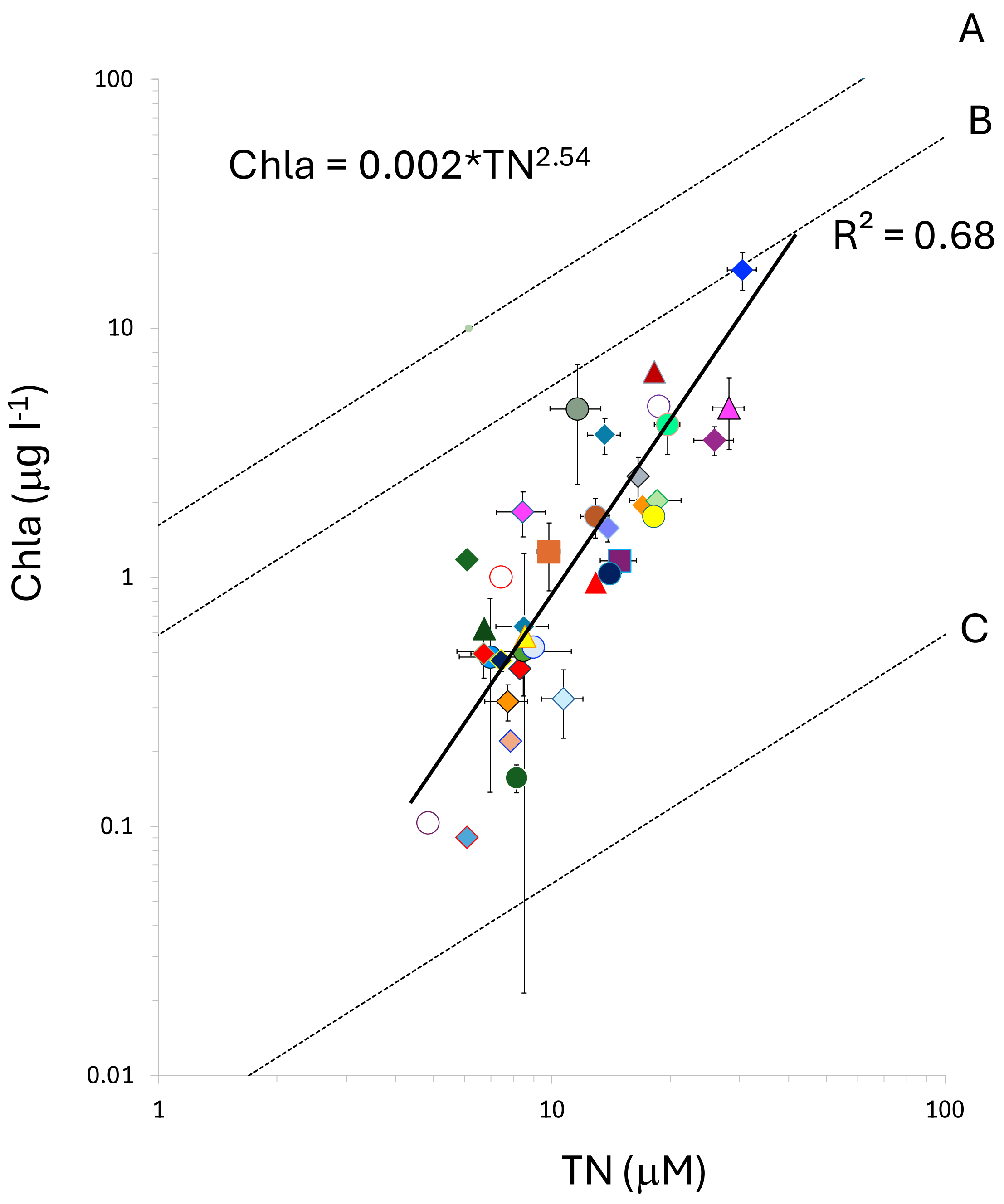

Figure S3. Patterns of chla vs. TN as eco-region means. Site symbols and reference lines follow Figure 1.

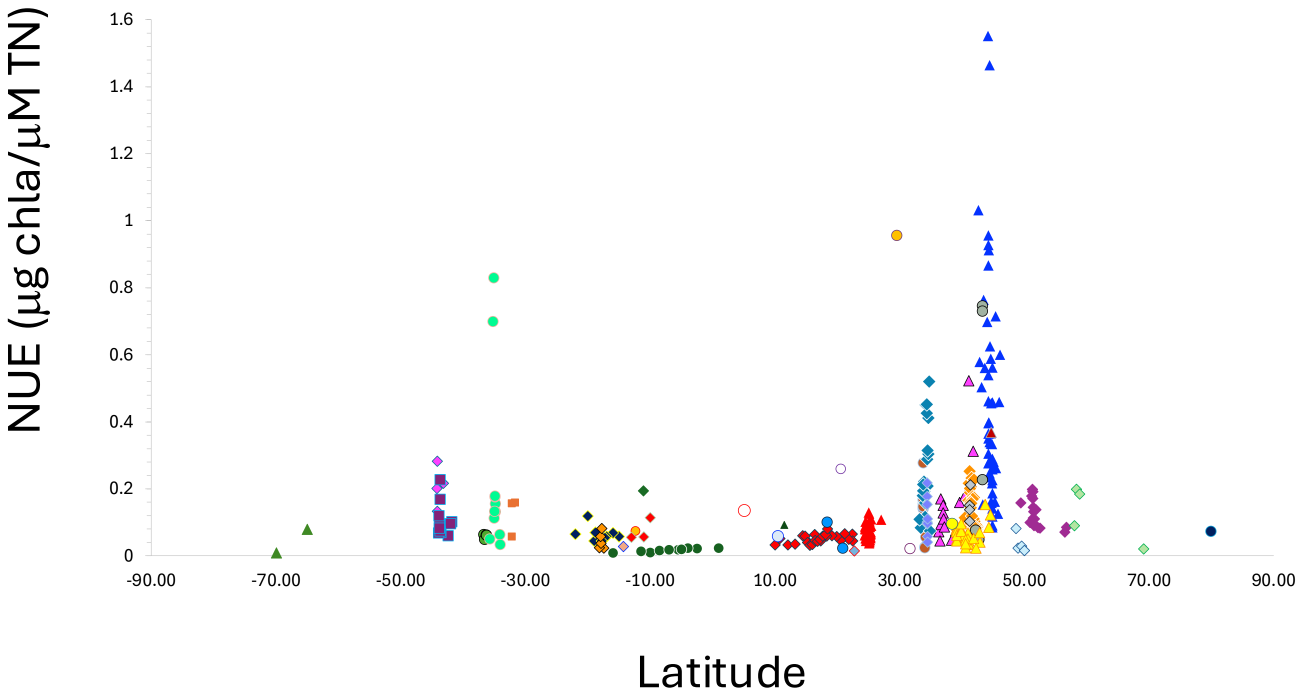

Figure S4. Variations in NUE as a function of latitude. Note low NUE values in low and high latitude systems. Southern Ocean sites (~-70º) is depicted as green triangles. Site symbols follow figure 1.

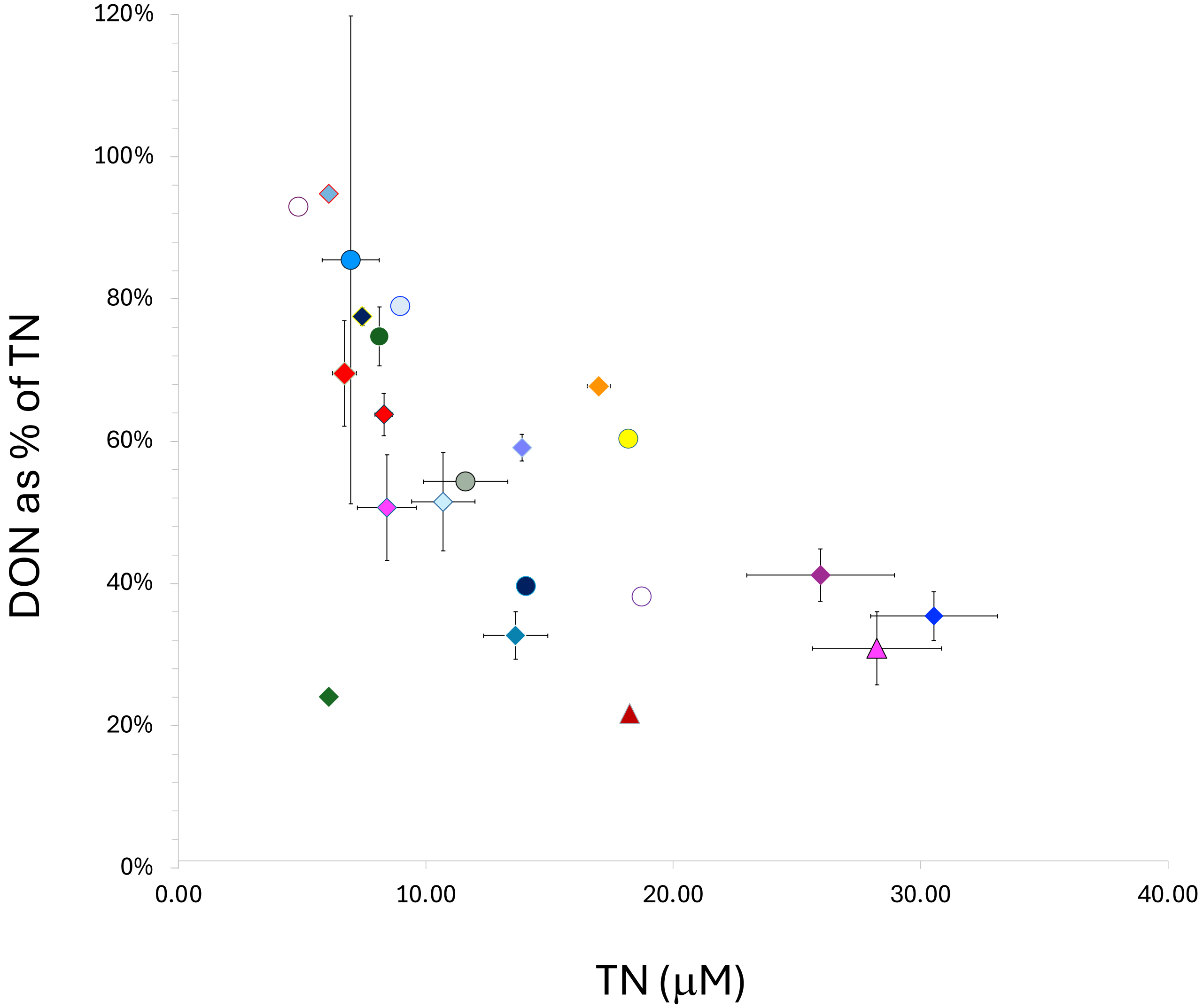

Figure S5. Cross-ecoregion variation in the contribution of DON to the TN pool as depicted by means and s.e. for each ecoregions. Ecoregions without error bars are represented by one site. Site symbols follow Figure 1.

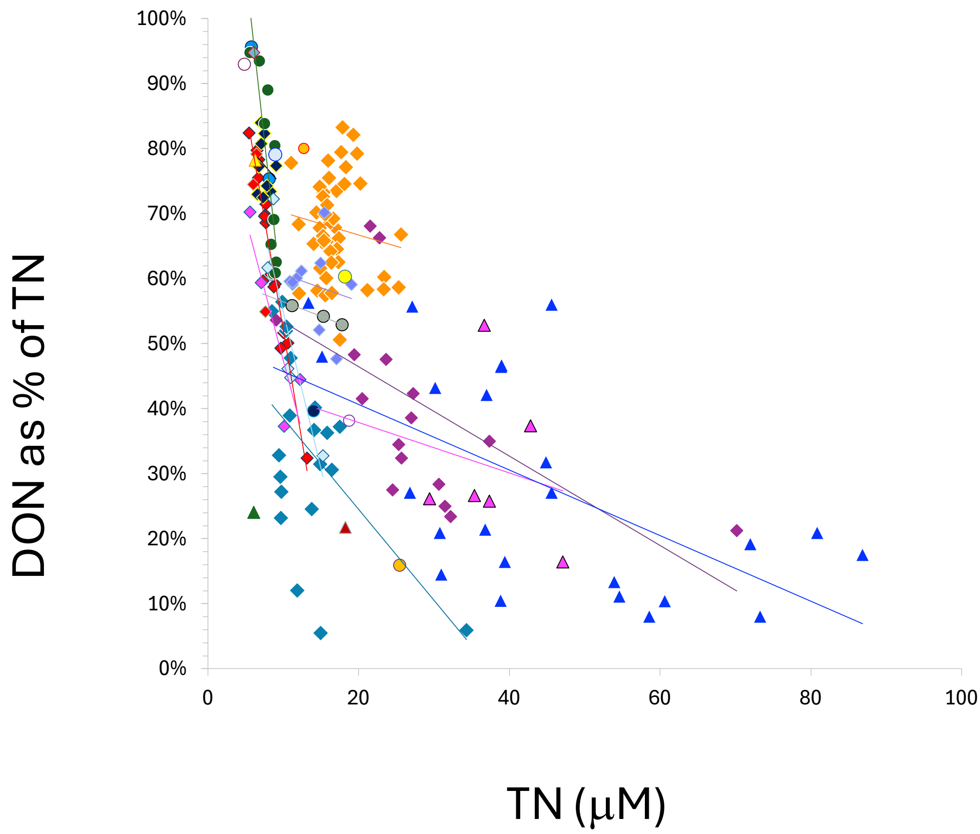

Figure S6. Same as figure 3 but with regression trend lines for ecoregions represented by multiple sites, highlighting the tendency for DON to decline with increasing TN across and within ecoregions.

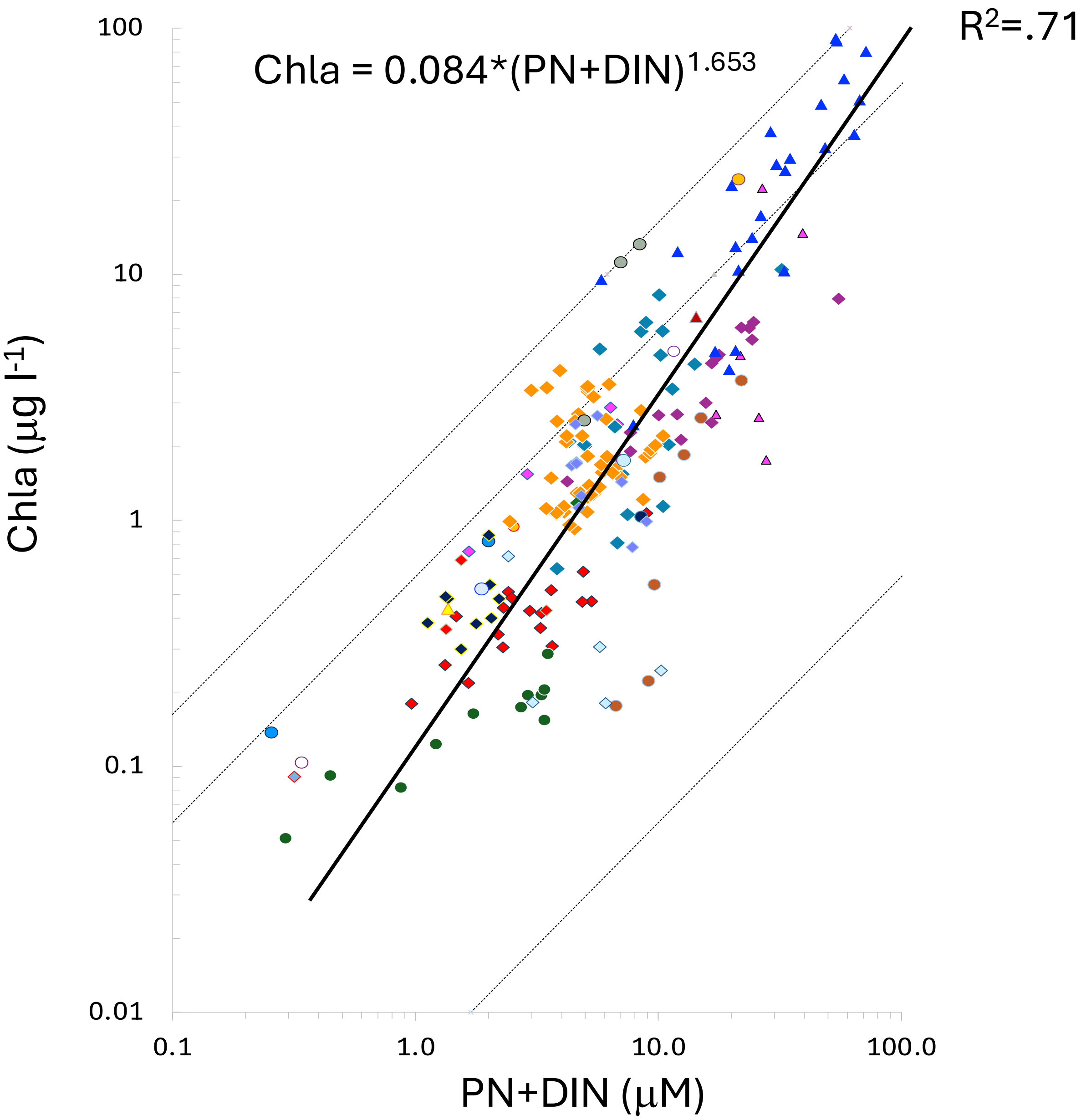

Figure S7. Chla as a function of PN+DIN (i.e. treating DON as a biologically-unavailable pool). The >1 scaling exponent (1.653) indicates that chla increases as an accelerating function of PN+DIN. Site symbols and reference lines follow Figure 1.

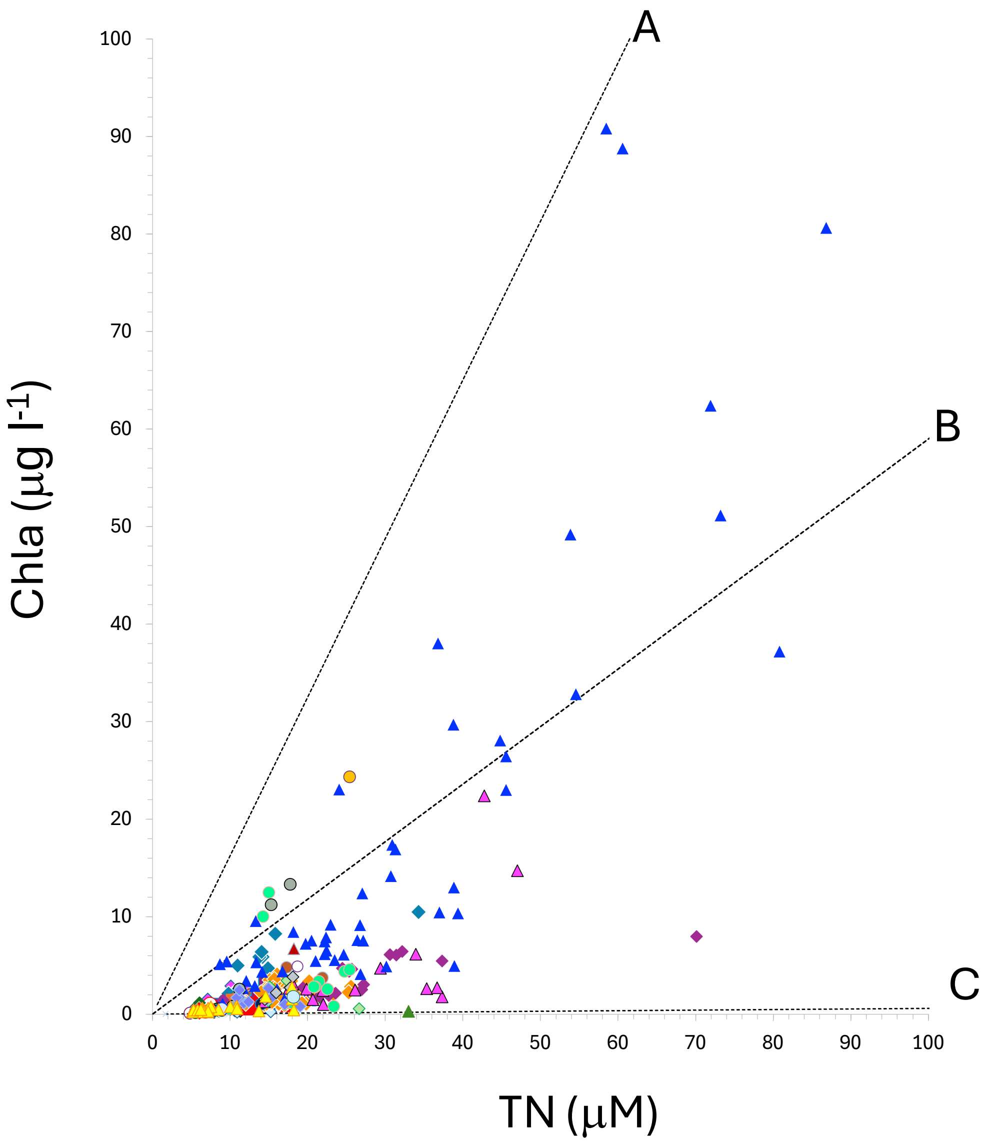

Figure S8. Cha vs. TN as depicted in figure 1 but plotted on linear scales. Reference lines and symbols follow figure 1.

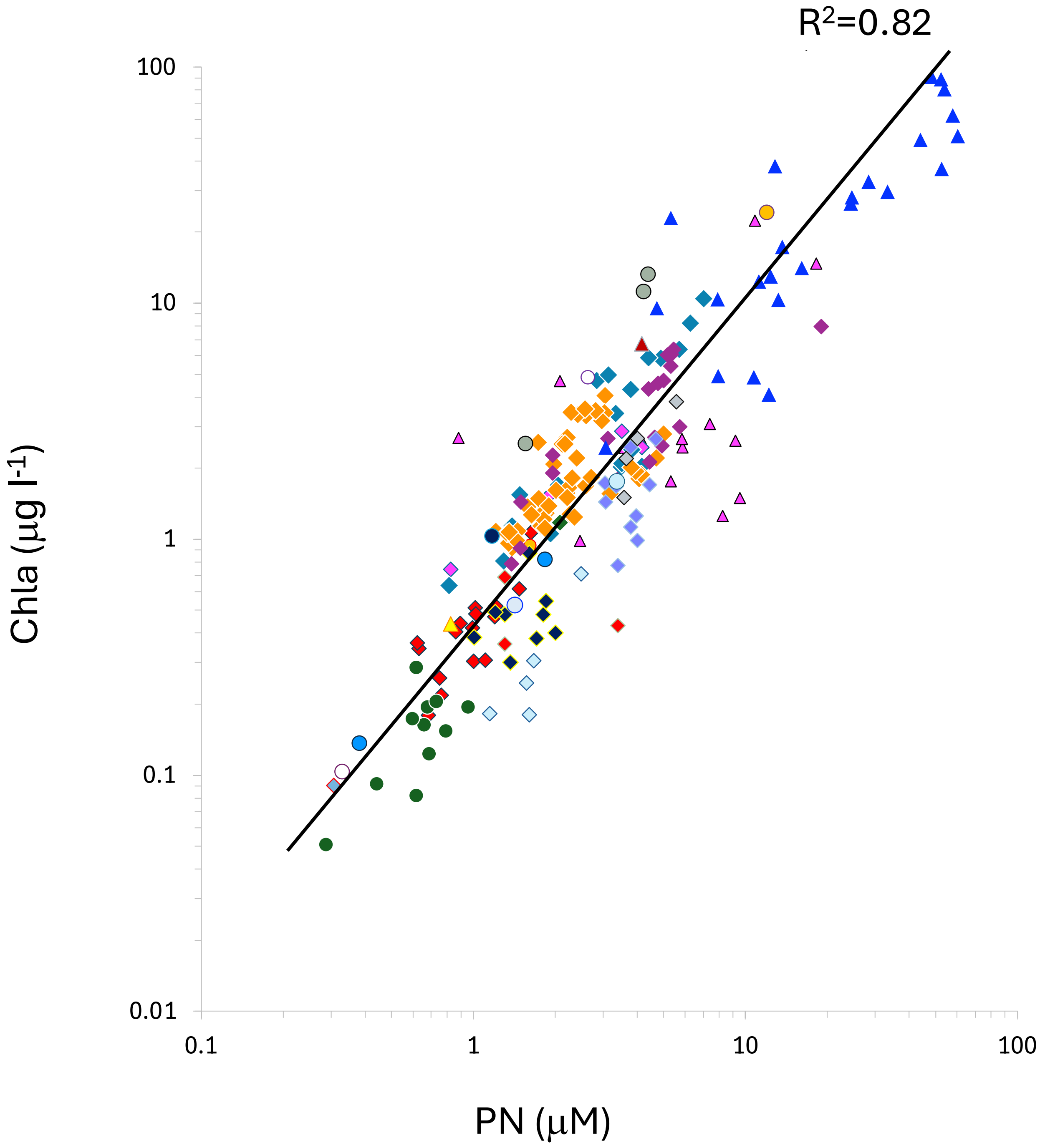

Figure S9. Stoichiometric pattern of surface chlorophyll and particulate nitrogen distribution across marine systems. Orthogonal regression line (log chlor = -0.382 + 1.40 * log PON) is depicted in black. Site symbols follow Fig 1.

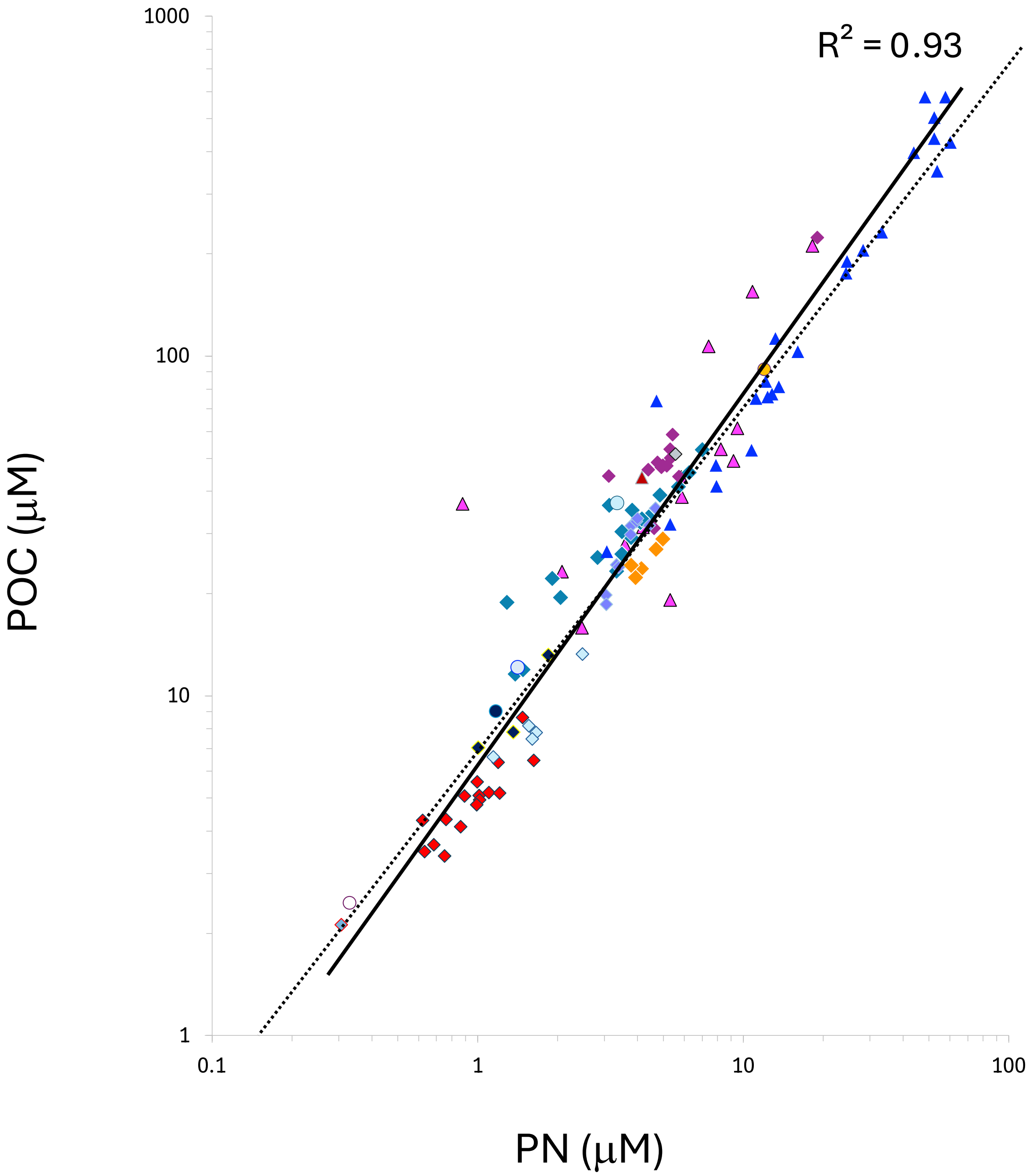

Figure S10. Stoichiometric pattern of surface particulate organic carbon and particulate nitrogen distribution across marine systems. Orthogonal regression line (log POC = 0.812 + 1.090 * log PN) is depicted in solid black. The dotted line depicted the Redfield ratio of 6.625:1.

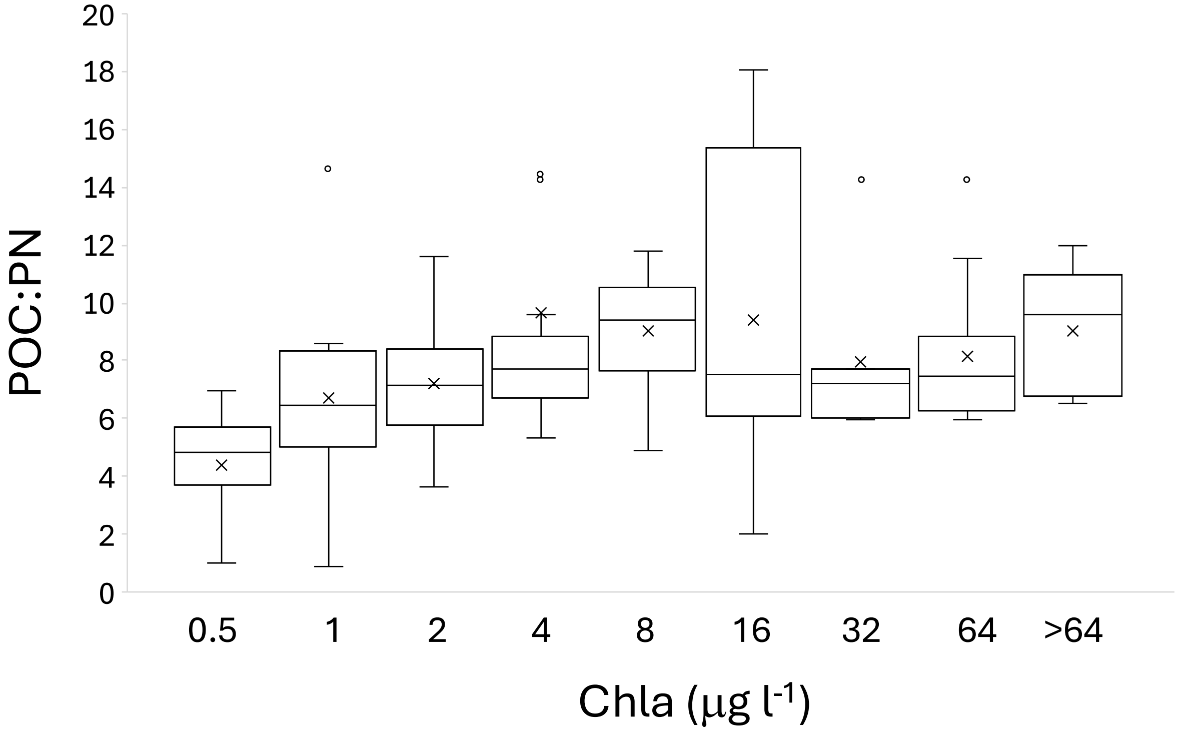

Figure S11. C:N ratio in particulate organic matter as arrayed by bins of increasing chla concentration to provide a more detailed perspective on the limited degree of variability in this ratio across gradients of phytoplankton biomass.

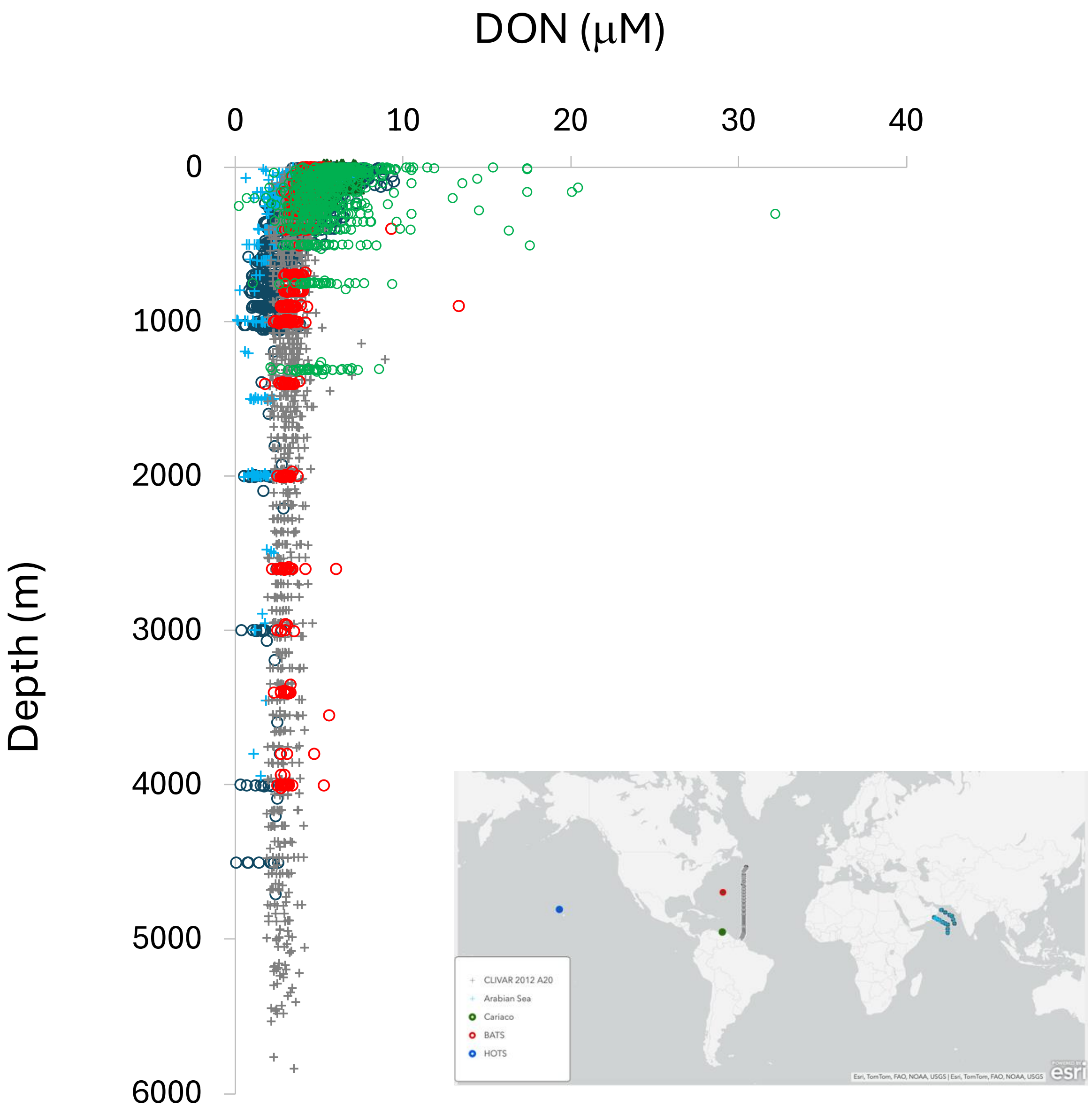

Figure S12. Example of depth profiles of DON for different regions of the ocean from time series and transect surveys

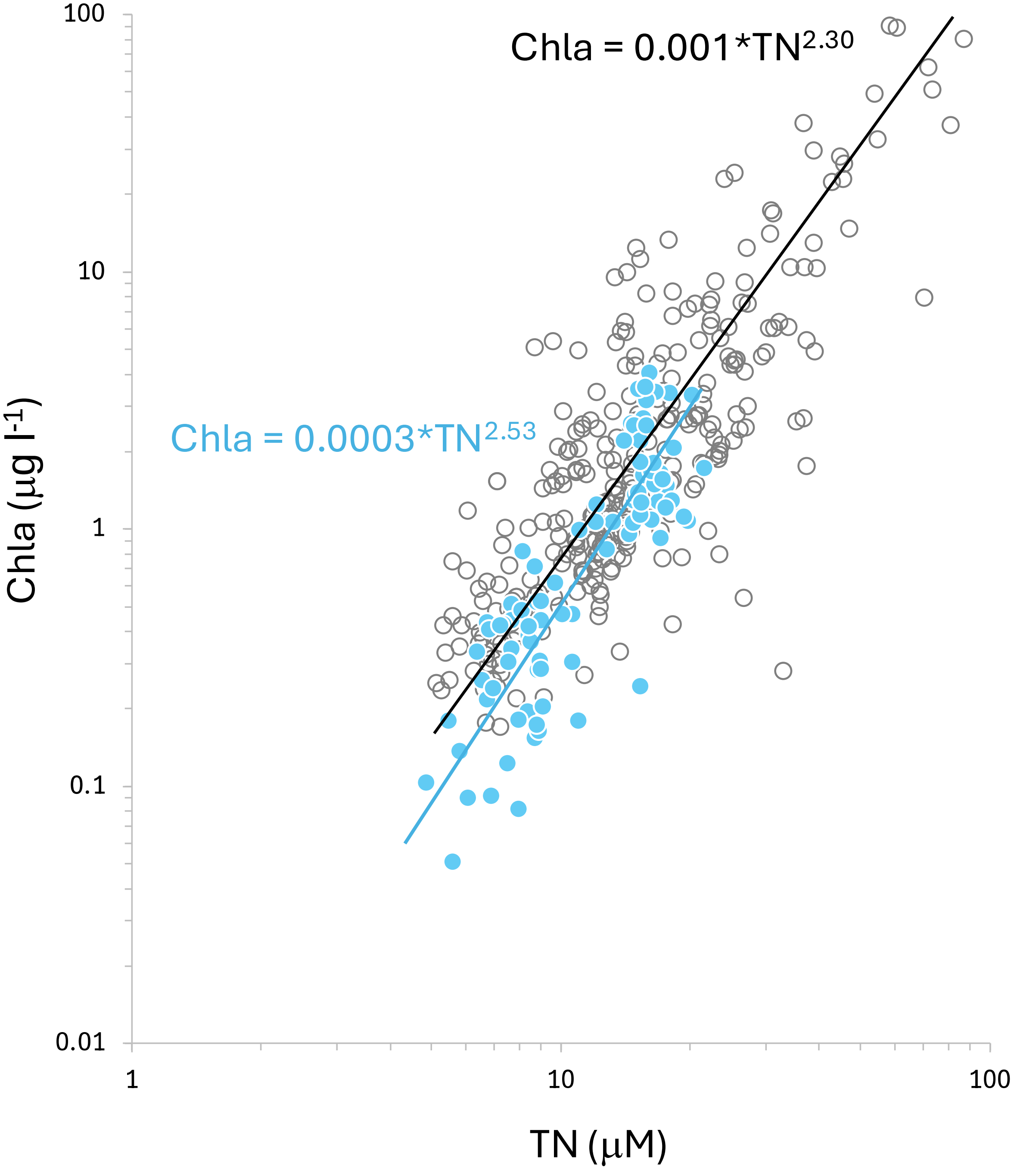

Figure S13. Patterns of chla vs. TN for open ocean (blue) and coastal (open) sites. Open ocean sites include: the Arabian Sea, Atlantic Subtropical Gyre, Canary Current, Caribbean Sea, Equatorial Pacific, Georges Bank, Gulf of Alaska, North Pacific Subtropical Gyre, Southern Ocean, see figure S1)
